# Interpersonal energy: New and bold directions in palliative care health professions education research

**DOI:** 10.1177/02692163231219949

**Published:** 2024-01-24

**Authors:** Laura Nimmon, Säde Stenlund

**Affiliations:** 1 The University of British Columbia, Faculty of Medicine, Centre for Health Education Scholarship, Department of Occupational Science and Occupational Therapy, Vancouver, BC, Canada; 2 The University of British Columbia, Faculty of Medicine, School of Population and Public Health, Vancouver, BC, Canada

*“Matter is organized energy and energy is disorganized matter – in the end, everything in our universe is energy”*

Connection between provider, patients, and families is powerful in end of life care. In this editorial, we propose the metaphor of interpersonal energy might be utilized to enhance human connection in the therapeutic relationship at end of life. We provide some contours to a definition of interpersonal energy that may deepen how we teach health professionals to engage in compassionate patient care. Our perspective joins a critical pedagogical turn in health professions education that strives to disrupt longstanding logics and ideologies that drive our educational systems and uphold status quo.^1^ Through such disruption, we can imagine new and bold directions in palliative care health professions education research. We don’t strive to have a singular and complete definition of interpersonal energy nor do we wish to constrain its meaning with rigid universal boundaries. Rather, we wish to stimulate a conversation that might elaborate the utility, shape the conceptualization, and nuance the meaning of interpersonal energy. Our aim is to ultimately deepen our understanding of the experience of connection that transcends ordinary understanding in palliative care and revitalize health professions education research.

The concept of interpersonal energy could support healthcare professionals meet the spiritual needs of patients and families at end of life. Spirituality in palliative care has widened to include a universal process of searching for meaning in connection with self and the world around us, and thus no longer refers to predominantly faith and religion.^2,3^ Despite its broad definition, dimensions of spiritual meaning-making are elusive and may often be rendered implicit, not explicit.^4^ These insufficient insights mean healthcare professionals might not be trained to appreciate the invisible subjective experiences encompassing an unconscious process of connectedness.^4^ As such, learners and healthcare professionals may struggle to meet the spiritual needs of patients and miss (or even dismiss) critical moments of connection that may have a positive impact on patients and families. For example, a mother describes how she “felt nurses’ non-physical presence”(p. 260) which helped her feel secure in the private last moments of her child’s life.^5^ Without an explicit way of understanding this non-physical presence, there is risk of dismissing such moments. We thus open the door to new research agendas and offer recommendations for palliative care health professions education researchers to explore the larger phenomenon of how individuals *experience* the presence of another person.

## The metaphor of energy

“Energy” is defined in the physical sciences as the capacity for doing work. It represents a force that causes movement and is studied and measured. Despite it being a well-studied phenomenon, energy remains an elusive concept. Richard Feynman (1918–1988), one of the most prominent physicists of the 20th century, claimed in his famous 1963 Lectures on Physics that “. . . in physics today, we have no knowledge of what energy is. . . It is an abstract thing in that it does not tell us the mechanism or the reasons for the various formulas”(p. 8).^6^ Some of the properties of energy are that energy is everywhere, it takes many forms, it cannot be created or destroyed, and it is transformed in various ways. Outside of the physical sciences, energy and vibration (vibes) are metaphors used anecdotally and persistently in the English language to convey subtle experiences of human interactions. For example, in a recent interview the lead choreographer for British Columbia Ballet vividly describes dancers interacting as “this invisible energy that is between us but vibrates in space.”^7^

We do not wish to prove or debate if energy exists between people, nor its physiological pathways or signals. Like unobservable elusive phenomena described in science like social power^8^ and interpersonal chemistry,^9^ the concept of interpersonal energy could be useful to capture significant subjective experiences in palliative care educational research. We distinguish our interest in energy as interpersonal energy which we describe as how an individual *experiences* the presence of another person. We thus borrow the concept from the physical sciences but do not propose that energy as it’s defined in the physical sciences and interpersonal energy are *literally* the same. Broadly and provisionally, it is subjective, not necessarily reciprocal, and it is phenomenological as it is an internal experience - an essence and meaning experienced by people. We use a metaphor of energy which is how this subjective experience *feels*. We do not conceptually constrain what it is by assuming cultural or contextual homogeneity. Energy may be experienced like two magnetic fields interacting or it may be sensed and internalized through impressions, feelings, and intuition in human interaction.

## Interpersonal energy in palliative care

Implicit references to interpersonal energy abound in participant narratives in the palliative care literature. These narratives suggest that experiencing interpersonal energy in its various dimensions may foster subjective spiritual, existential, and embodied experiences. For example, although people with advanced cancer identify kindness, genuineness, and honesty as sources of compassionate response, they cite love most frequently when distinguishing compassion from sympathy and empathy. One patient describes “compassion I think means to me. . .giving me unconditional love.”^10^ (p. 444) We suggest positive interpersonal energy can create a subjective feeling of love, tranquility, and belonging, whereas experiences of negative energy can lead to fear, stress, and loneliness. Social experiences of energy can be similar (mutually uplifting) or different (comforting to one individual, depleting the other). One thread that remains steady in our conceptualization is that interpersonal energy represents a profoundly subjective individual experience. It can hold subjective positive and/or negative dimensions or perhaps invoke some other experience that is neither positive nor negative. Thus, it is not always causal nor symmetrical. Certainly, this initial conceptualization is nascent and might need to encompass transcendent meaning that surpasses the boundaries of reality, such as the experience of sensing the presence of a deceased loved one described by many participants in palliative care research.^4^ Although the concept is emergent, it generates preliminary novel health professions education research questions.

## Revitalizing health professions education research

We are curious about several health professions education research questions: How might learners be fully present in a nonverbal but meaningful way to connect with a dying patient, and is this less taxing for the patient than engaging through oral communication? Building on the work of Lavecchia et al.,^11^ should patients’ subjective experiences of interpersonal energy be considered meaningful measures when evaluating learners who engage in serious illness conversations? How can learners develop skillful perception and heightened sensory awareness of patients, family members, and colleagues’ interpersonal energy? Are there motivations, actions, and behaviors that can be taught that can foster a positive and soothing experienced energy for patients in the provision of compassionate palliative care?

The construct raises fascinating broader ethical questions. Reflecting on the work of Ritchie and Kotwal,^12^ should learners be attuned to a dying patient’s degree of loneliness and their need for comforting interpersonal energy? We are also curious how to support learners to protect themselves if they are highly attuned to interpersonal energy and experience certain interactions as overwhelming and depleting. Building on the work of Kumagai and Naidu,^13^ we wonder what is the reflective practice of interpersonal energy that meets ethical and professional responsibilities? Jung’s^14^ work stimulates us to ponder if learners interpretations of a patient, family member, or team member’s energy is partly a reflection of their internal state. Such new health professions education questions about interpersonal energy are suited to qualitative methodologies with their rich descriptions of context, personal meaning, emotional and social nuances, and insight into unrevealed thoughts and layers of detail.^15^

## Igniting a new conversation

A revitalized language for interacting with the dying might enhance how learners and healthcare professionals respond to the spiritual needs of patients and families. Death and dying present a unique context where spirituality as a dynamic dimension of human life may be intensified and its meaning deeply personal. Sensitive and tentative relational approaches are needed to capture how individuals and community experience, express and/or seek meaning, purpose, and transcendence, and the way they connect to moment, to self, to others, to nature, to the significant, and/or the sacred.^3^ We suggest the elusive but powerful experience of interpersonal energy may have the potential to support the wellbeing of patients, families, and healthcare professionals as they process subtle sensations that may have an unspoken spiritual and transcendent quality. We offer a preliminary attempt to define and provide scope for a hidden aspect of social communication that enables us to ask original educational research questions for palliative care.

We believe palliative medicine could widen its lens on the nature of human interaction and consider the terminology and concept of interpersonal energy that as human beings we intuitively, covertly, or even unconsciously experience. Certainly, we hope that by introducing interpersonal energy as a concept we might enable a different educational research gaze and expand how we consider best care to holistically serve dying patients and their families in the context of a therapeutic interpersonal relationship. Palliative care has made efforts to embrace the depth of human meaning-making and broadly defines spirituality as constituting multidimensional character.^3,4^ Yet, despite its broad definition, we may have overlooked delicate aspects of how humans engage in conscious or unconscious processes of spiritual meaning-making through connection to self and other. We invite colleagues to expand, deconstruct, or distill our meaning, and we welcome skeptics to challenge, unsettle, and enrich it.

Advancing our understanding of the subjective experience of interpersonal energy promises expansion of consciousness around what constitutes relationship centered compassionate end of life care. This metaphor may help learners and healthcare professionals support patients and loved ones who grapple with deeply existential experiences and questions around connection and felt presence that are challenging to articulate, or are criticized and dismissed.^4^ Furthermore, introducing interpersonal energy into medicine and healthcare’s understandings and vocabularies might be especially important in an era where we face creeping dehumanization because of electronic medical records, AI, telemedicine, time, and resource constraints. We are at an inflection point in health professions education where our learners need to be trained to engage in the highest levels of nuanced forms of compassionate communication.^16^ We thus need to make efforts to be *more* human in the communication approaches we teach learners.^16^ The metaphor could legitimize learners and healthcare professionals’ holistic embodied nature and potential as healers, nuancing dominant conceptualizations of technical analytic curers.^17^ Perhaps most importantly, interpersonal energy with its vast potential in the context of death and dying might be an important contribution toward advancing our understanding of the nature of healing and bring universal significance to our search for connection and meaning.
